# Hyperbilirubinemia and neurologic signs in dogs with non-associative immune-mediated hemolytic anemia: 81 cases (2015–2024)

**DOI:** 10.1093/jvimsj/aalaf034

**Published:** 2026-01-21

**Authors:** Sasha Chapman, John M Angles, Christine Griebsch, Jane Yu

**Affiliations:** Animal Referral Hospital Canberra, Internal Medicine Department, Pialligo, Australian Capital Territory, 2609, Australia; Animal Referral Hospital Canberra, Internal Medicine Department, Pialligo, Australian Capital Territory, 2609, Australia; Sydney School of Veterinary Science, Faculty of Science, The University of Sydney, Sydney, New South Wales 2006, Australia; Animal Referral Hospital Canberra, Internal Medicine Department, Pialligo, Australian Capital Territory, 2609, Australia; Department of Veterinary Clinical Sciences, Jockey Club College of Veterinary Medicine and Life Sciences, City University of Hong Kong, Kowloon Tong, Hong Kong, China

**Keywords:** kernicterus, neurologic, plasmapheresis, prognosis

## Abstract

**Background:**

Bilirubin encephalopathy is a poorly recognized complication in dogs with immune-mediated hemolytic-anemia (IMHA).

**Hypothesis/Objectives:**

Assess serial trends of hyperbilirubinemia and the association between bilirubin concentrations and neurologic signs in dogs with IMHA.

**Animals:**

Eighty one dogs with non-associative IMHA and hyperbilirubinemia.

**Methods:**

Multicenter retrospective cohort study. The signalment, clinical signs, clinicopathological data, treatment, and outcome were evaluated. Bilirubin concentrations were recorded at the baseline, peak, initial decrease, and normalization. Univariable logistic regression was used to determine the association between neurologic signs and hyperbilirubinemia.

**Results:**

The median bilirubin concentrations at the baseline, peak, and initial decrease were 2.5 (IQR, 1.4–4.8), 3.7 (IQR, 1.8–24.2), and 1.1 mg/dL (IQR, 0.5–4.3; 43, 64, and 19 μmol/L), respectively. Twenty percent (16/81) of dogs developed neurologic signs. Neurologic signs included stupor, non-ambulatory tetraparesis, and generalized seizures. A significant association was found between the presence of neurologic signs and the baseline, peak, and fold-change of bilirubin concentration (*P* < .001). The odds of having neurologic signs were 12.2 (95% CI, 3.1-48.2) for dogs with baseline bilirubin concentrations ≥3.3 mg/dL (≥57.5 μmol/L), and 93.3 (95% CI, 11.0-795.5) for dogs with peak bilirubin concentrations ≥13.9 mg/dL (≥239.5 μmol/L).

**Conclusions and clinical importance:**

Although the causation of the neurologic signs cannot be attributed solely to bilirubin based on our study, these findings emphasize the importance of monitoring serum bilirubin concentrations and the development of neurologic signs in dogs with IMHA. The results reflect findings in our study population and may not be directly applicable to all dogs with IMHA.

## Introduction

Immune-mediated hemolytic anemia (IMHA) is a common autoimmune disease in dogs caused by the immunoglobulin or complement coating or both of red blood cells (RBC). This binding leads to intravascular lysis of RBC, resulting in hemoglobinemia, extravascular phagocytosis or both and subsequent prehepatic hyperbilirubinemia.^1^ Mortality rates of 26%–70% have been reported.^2–7^

Hyperbilirubinemia has been reported in 37%–82.5% of dogs with IMHA.^3^^,^^4^^,^^6^^,^^8^ Higher bilirubin concentrations have been associated with an increased risk of IMHA relapse^9^ and bilirubin concentrations >1.5^3^ and 5.0 mg/dL^4^ (25.5 and 85.5 μmol/L) have been associated with increased mortality, with overlap between survivors and non-survivors.^10^

Neurologic signs in dogs with IMHA are reported rarely but often are ascribed to ischemia, thromboembolism, or ischemic stroke; acute bilirubin encephalopathy (ABE); or inflammatory encephalitis.^8^ In both people and dogs, IMHA is associated with a hypercoagulable state that frequently leads to thrombosis, most notably pulmonary thromboembolism.^11^ Cerebrovascular events in thrombocytopenic dogs with IMHA have been described.^12^^,^^13^ Although transient cerebral ischemia or ischemic strokes may contribute to neurologic signs, ABE is increasingly being recognized in dogs with severe hyperbilirubinemia and acute onset neurologic dysfunction.^14–17^

Acute bilirubin encephalopathy is an uncommon complication of hyperbilirubinemia reported in people and is rarely reported in veterinary medicine.^15^ Acute bilirubin encephalopathy, or its chronic form, kernicterus, describes both the pathologic finding of bilirubin-induced yellow staining of the deep nuclei of the brain and the associated secondary neurologic compromise.^18^^,^^19^ In people, ABE often occurs in newborns, resulting in decreased feeding, hypotonia or hypertonia, high-pitched cry, opisthotonus, seizures, and death.^20^ Permanent impairments can occur if bilirubin concentrations are not rapidly decreased.^21^ Although unconjugated bilirubin concentration is a predictive risk factor for developing ABE, other pathophysiologic mechanisms likely exist.^14^^,^^22^

Bilirubin concentrations in dogs with IMHA and ABE vary between 35.3 and 90.7 mg/dL (609–1563 μmol/L) and have been associated with a grave prognosis.^14–17^ This condition can result in a comatose state, refractory seizures, and cardiopulmonary arrest.^14^ Although the bilirubin concentration is a prognostic indicator of survival in dogs with IMHA, its association with neurologic signs has not been explored. Identifying threshold bilirubin concentrations above which neurologic signs could be expected may facilitate diagnosis of this potentially fatal complication and enable early intervention with treatments, such as therapeutic plasma exchange (TPE), which may decrease immunoglobulin and bilirubin concentrations.^15^

We aimed to characterize the serial trends of hyperbilirubinemia in dogs with IMHA and to investigate the association between bilirubin concentrations and the presence of neurologic signs. The second objective was to report the outcome of dogs with neurologic signs. The hypothesis was that the presence of neurologic signs in dogs with IMHA is associated with the magnitude of hyperbilirubinemia.

## Materials and methods

### Case selection

Medical records from 8 veterinary referral institutions across Australia were retrospectively reviewed for cases of IMHA diagnosed between January 2015 and December 2024. Terms used during the medical record search included “immune-mediated hemolytic anemia,” “IMHA,” “hyperbilirubinemia,” “acute bilirubin encephalopathy,” “ABE,” and “kernicterus.” Cases were classified as either “diagnostic” or “supportive” of IMHA based on the American College of Veterinary Internal Medicine (ACVIM) consensus guidelines.^23^ Inclusion criteria for the “diagnostic” group included anemia (packed cell volume [PCV], <37%), at least 2 signs of antibodies directed against RBC (ie, spherocytosis, positive saline agglutination test [SAT], or a positive direct antiglobulin test [DAT]) or a positive SAT that persisted after saline washing of RBCs. A second group was included for analysis and was considered “supportive” if they had anemia, at least 1 sign of antibodies directed against RBCs or a positive SAT that persisted with RBC saline washing, and hyperbilirubinemia. Only dogs with hyperbilirubinemia (total serum bilirubin concentrations >0.9 mg/dL [>15 μmol/L], reference interval [RI] 0–0.9 mg/dL per Vetnostics pathology, Australia) and at least 2 measured total bilirubin concentrations with 1 determination at initial presentation were included. The DAT and the presence or absence of auto-agglutination were assessed on pre-treatment and pre-transfusion blood samples. The SAT protocol was performed using a standard dilution of 4 drops of saline to 1 drop of blood.^23^

Only cases classified as “non-associative IMHA” were included^23^ based on the exclusion of underlying causes. All dogs underwent either thoracic radiography and abdominal ultrasonography, or whole body computed tomography (CT). Ancillary diagnostic tests were performed at the discretion of the attending veterinarian. These included urinalysis, echocardiography, a coagulation profile (ie, activated partial thromboplastin time and prothrombin time with or without thromboelastography), heartworm testing, titers for rickettsial diseases, leptospirosis PCR and antibody testing, fecal parasitology or enteropathogen testing, and *Neospora caninum* serology.

Cases were excluded if the medical record was incomplete or if any of the following conditions were diagnosed or suspected: hepatic or post-hepatic, or both, causes of hyperbilirubinemia, including pancreatitis causing extrahepatic biliary duct obstruction based on CT or ultrasonographic examination, sepsis, advanced renal disease (International Renal Interest Society acute kidney injury stages III, IV, or V^24^) gastrointestinal disease, pre-existing neurologic disease, or concurrent thrombocytopenia (platelet count < 50 000 × 10^9^/L).

Documentation of a complete neurologic examination performed by an internal medicine specialist or resident was required for inclusion in the cohort of dogs described as neurologically abnormal. At minimum, the neurologic examination included an assessment of mentation, gait, cranial nerves, postural reaction testing, and spinal reflexes. The dog also had to be assessed by the primary clinician to have severe neurologic dysfunction beyond general malaise or decreased mentation.

### Data collection

Total serum bilirubin concentrations were extracted from medical records at 4 time points (Table 1). Serum biochemistry analysis methods varied and included both in-house and external laboratory testing. The in-house and external serum bilirubin concentrations were measured using the colorimetric diazo method, which is known to be minimally affected by hemolysis.^25^ The time at which follow-up blood samples were collected was not standardized because of the retrospective nature of the study. The severity categories of hyperbilirubinemia were established based on a previous study^26^ and the RIs of external laboratories used in our study. Hyperbilirubinemia was categorized as mild (>0.9–2.9 mg/dL; >15–50 μmol/L), moderate (>2.9–5.8 mg/dL; >50–100 μmol/L), severe (>5.8–11.60 mg/dL; >100–200 μmol/L), and very severe (>11.60 mg/dL; ≥200 μmol/L).

**Table 1:** Time points at which serum bilirubin concentrations from dogs with hyperbilirubinemia associated with IMHA were collected.

Time point	Description
**Baseline**	Initial presentation to a veterinarian (>0.9 mg/dL; >15 μmol/L)
**Peak**	Highest recorded serum bilirubin
**Decrease**	Initial decrease in serum bilirubin by at least 1.0 mg/dL (17 μmol/L)^43^
**Normalization**	Normalization of serum bilirubin (<0.9 mg/dL; <15 μmol/L)

Abbreviation: IMHA = immune-mediated hemolytic-anemia.

Survival to discharge, death, or euthanasia was recorded. Data including age, breed, neuter status, pre-existing comorbidities, clinical signs, neurologic signs, time to resolution of neurologic signs, outcome, time to discharge, reason for euthanasia or death, immunosuppressive treatments, and other treatments were recorded. Serum biochemistry and CBC results were recorded at initial presentation.

Blood transfusions received for the management of IMHA were recorded and included the date or dates of transfusion, the blood product, major cross match compatibility, and transfusion reactions. All cross matching was performed utilizing a validated patient-side major cross matching kit (Quick Test XM Canine, Alvedia, Lyon, France).

### Statistical analysis

Statistical analysis was performed using R studio.^27^ For continuous variables, normality was assessed by vizualization of histograms. The independent *t*-test was used for parametric data and the Mann–Whitney U test was used for non-parametric data. The mean and SD were reported when data were normally distributed. The median, IQR, and range (minimum to maximum) were reported when data were not normally distributed. Categorical variables were compared using chi-squared tests, with count and percentage reported.

The discriminatory ability of baseline bilirubin, peak bilirubin, decreasing bilirubin, and fold change (defined by peak bilirubin divided by baseline bilirubin) were assessed using receiver operating characteristic (ROC) curves with optimal cut-offs to predict neurologic signs. The optimal cut-offs were further evaluated by univariable logistic regression modeling. The association between neurologic signs and baseline, peak, and decreasing bilirubin, fold change, time to reach peak bilirubin, age, sex, PCV at initial presentation, serum creatinine and blood urea nitrogen (BUN) concentration, alkaline phosphatase activity, neutrophils, monocytes,^28^ and blood transfusion were assessed using univariable logistic regression modeling.

## Results

The initial search identified 541 dogs diagnosed with IMHA. Cases were excluded when there was an absence of at least 2 documented bilirubin concentrations with 1 at initial presentation (*n* = 304), documented associative disease (*n* = 60), lack of hyperbilirubinemia (*n* = 53), concurrent thrombocytopenia (*n* = 23), incomplete medical records (*n* = 12), advanced renal disease (*n* = 5), and extra-hepatic biliary duct obstruction (n = 3). A total of 81 cases were eligible for inclusion and were classified as either “diagnostic” (*n* = 73, 90%) or “supportive” (*n* = 8, 10%) of IMHA based on the recent ACVIM consensus guidelines.^23^ The median PCV at initial presentation was 17% (IQR, 14–23; range, 6%–33%). Saline agglutination testing was performed in all 81 dogs by the attending clinician. A DAT (Coombs test) was available for 35 dogs and was positive in 21 dogs and negative in 14 dogs. The degree of spherocytosis as interpreted by a board-certified clinical pathologist was available in 78/81 dogs. Ghost cells were reported in 9/78 cases (Table 2).

**Table 2:** Signalment and clinicopathologic features of 81 dogs with non-associative IMHA.

Categorical variables	Number of dogs (%)		
**IMHA classification**	Diagnostic: 73 (90%) Supportive: 8 (10%)		
**Saline agglutination test**	Positive: 78 (96%) Negative: 3 (4%)		
**Direct antiglobulin test (DAT)^a^**	Positive: 21 (60%) Negative: 14 (40%)		
**Degree of spherocytosis^b^**	Mild (1+): 17 (22%) Moderate (2+): 50 (64%) Severe (3+): 11 (14%)		
**Ghost cells^b^**	Positive: 9 (12%) Negative: 69 (88%)		
**Sex distribution**	Female neutered: 36 (44%) Female entire: 8 (10%) Male neutered: 37 (46%)		
**Continuous variables**	Median	IQR	Range
**Age at admission**	7 years	5–10	9 months to 16 years
**PCV at presentation**	17%	14–23 (RI 37%–55%)	6%–33%

^a^DAT was only available in 35 dogs.

^b^Information about the degree of spherocytosis and ghost cells was available in 78 dogs.

Abbreviations: IMHA = immune-mediated hemolytic-anemia; PCV = packed cell volume.

Twenty-nine breeds were included (Table S1). The age of the dogs at admission ranged from 9 months to 16 years (median, 7 years; IQR, 5–10). Eight (10%) were intact females, 36 (44%) were spayed females, and 37 (46%) were neutered males.

Immunosuppressive and thromboprophylactic treatments are listed in Table 3. Supportive treatment included IV fluids, antibiotics (amoxicillin-clavulanate and metronidazole), analgesia (methadone), anti-emetics (maropitant, ondansetron, and metoclopramide), and gastroprotectants (omeprazole, misoprostol, or sucralfate). Human intravenous immunoglobulin (hIVIG) was given adjunctively to 1 dog, whereas another dog received 3 sessions of TPE. This dog experienced the resolution of neurologic signs and survived to discharge.

**Table 3:** Immunosuppressant and thromboprophylactic treatments administered to 81 dogs with IMHA and hyperbilirubinemia**.**

Medication	Number of dogs	Median dose (mg/kg/day)	IQR (mg/kg/day)	Range (mg/kg/day)
**Immunosuppressive treatment**				
** Prednisolone**	77	2	1.9–2.3	1.0–3.9
** Dexamethasone**	64	0.2	0.20–0.3	0.15–0.57
** Cyclosporine**	46	5.32	4.5–6.9	2.3–17.2
** Azathioprine**	15	1.9	1.5–2.0	1.0–2.1
** Leflunomide**	2	2.44	2.3–2.6	2.17–2.7
** Mycophenolate mofetil**	3	17	15.8–22.0	14.6–27.0
**Thromboprophylaxis treatment**				
** Clopidogrel**	64	2.59	2.0–3.2	0.49–7.5
** Enoxaparin**	29	1.0	0.8–1.0	2.4–2.0
** Rivaroxaban**	13	1	0.8–1.1	0.5–5.0

Abbreviation: IMHA = immune-mediated hemolytic-anemia.

Of the 81 dogs, 10 had no blood products administered, 22 received a single blood transfusion, and 49 dogs received multiple blood transfusions (Table 4). The median number of blood transfusions administered was 2 (IQR, 1–3; range, 0–13). Forty-two (59%) dogs received packed RBCs (pRBCs), 14 dogs (17%) received stored whole blood, 6 dogs received fresh whole blood (7%), and 9 dogs (13%) received a combination of stored or fresh whole blood or pRBCs. All dogs that received a blood transfusion were either cross match compatible in the first instance or had cross match compatibility testing for blood products administered 72 hours after their first non-crossed matched transfusion, consistent with recommendations.^29^ In total, 21 transfusion reactions were reported. Seventeen of 71 dogs (24%) had 1 and 4/71 dogs (6%) had 2 transfusion reactions. The reported transfusion reactions all were classified as mild and non-life threatening, resolving after a decrease in transfusion rate. They included allergic transfusion reactions (*n* = 17), febrile non-hemolytic transfusion reactions (*n* = 3), and transfusion-associated circulatory overload (*n* = 1).^30^

**Table 4:** Number of blood transfusions administered to 81 dogs with IMHA and hyperbilirubinemia.

Number of blood transfusions administered	Number of dogs
**0**	10
**1**	22
**2**	24
**3**	10
**4–5**	9
**6–7**	3
**8–13**	3

Abbreviation: IMHA = immune-mediated hemolytic-anemia.

### Neurologic complications in dogs with IMHA

Sixteen dogs (20%) had neurologic signs. A tentative diagnosis of ABE was assigned by the primary clinician because of a combination of substantially increased bilirubin concentration, compatible neurologic signs, and the absence of other metabolic causes for neurologic signs. Advanced imaging of the brain was considered in 2 cases but not performed.

**Figure 1:**
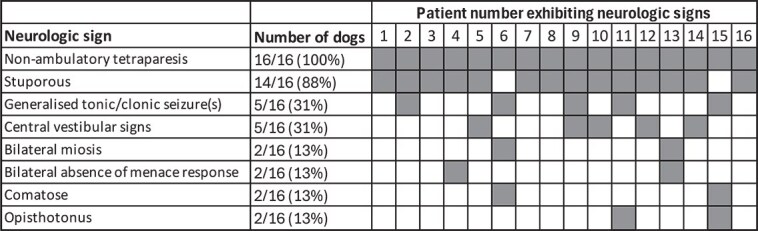
Neurological signs in 16 dogs with IMHA, hyperbilirubinemia and suspected bilirubin encephalopathy. Abbreviation: IMHA = immune-mediated hemolytic-anemia.

Neurologic signs are listed in Figure 1. The most common neurologic signs reported were non-ambulatory tetraparesis (16/16; 100%) and stupor (14/16; 88%). The reported seizures (*n* = 5) were all generalized (tonic/clonic). Two animals had 2 seizure events, 2 had a single isolated seizure, and 1 had 4 seizures. In dogs with multiple seizure events, all events occurred within a 24-hour period and at least 2 hours apart. Central vestibular signs were variable but consisted of altered mentation; proprioceptive deficits ipsilateral to the side of the lesion; rotary, horizontal, or vertical nystagmus; and, ipsilateral head tilt toward the side of the lesion. Animals with bilaterally absent menace responses retained normal vision with intact pupillary light reflexes. Bilateral miosis occurred in 2 animals, was transient, and accompanied by other neurologic deficits, making primary ocular disease unlikely.

In all dogs, peak bilirubin concentrations occurred within 24 hours of the recorded onset of neurologic signs. Only 1 dog was presented with neurologic dysfunction at admission, whereas the remaining 15 developed neurologic signs after a median hospitalization period of 2 days (IQR, 1–3; range, 1–6). All neurologic signs developed acutely, but the clinical course leading up to the acute deterioration was not always available. A decrease in bilirubin concentration coincided with reported improvement in neurologic signs in the 5 dogs that survived. For all survivors, neurologic signs resolved within a median of 5.5 days (IQR, 3.5–7.8; range, 2–10).

### Association between bilirubin concentrations and neurologic signs

**Figure 2:**
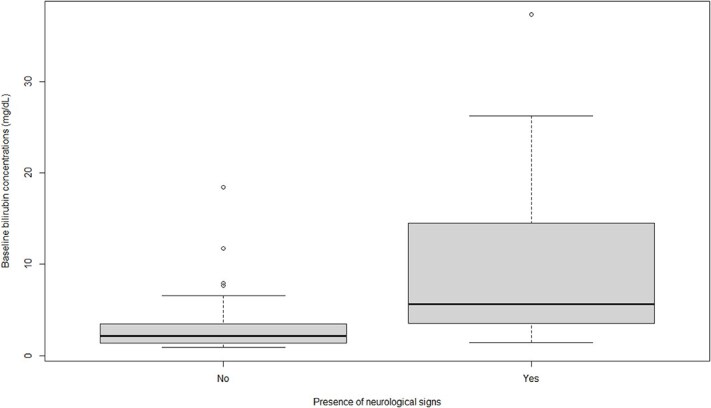
Boxplot showing the difference in baseline bilirubin concentrations at initial presentation in 16 dogs with neurological signs and 65 dogs without neurological signs. Boxes represent the IQR from the 25th and 75th percentile. The horizontal bar in each box represents the median value. Outliers are represented by dots.

Baseline bilirubin concentrations were significantly associated with the presence of neurologic signs (*P* = .001; Table 5). The higher the bilirubin concentration, the higher were the odds of developing neurologic signs (Table 6; Figure 2).

**Table 5:** Association between bilirubin concentrations and neurologic signs in 81 IMHA dogs.

	Dogs with neurologic signs			Dogs with no neurologic signs			*P*-value
	Median	IQR	Range	Median	IQR	Range	
**Baseline bilirubin mg/dL (μmol/L)**	5.6 (96.5)	3.6–13.1 (62.1–225.9)	1.4–37.3 (24.1–643.1)	2.1 (37)	1.3–3.5 (22.4–60.3)	0.9–18.4 (15.5–317.2)	.001
**Peak bilirubin mg/dL (μmol/L)**	40.9 (706)	35.0–56.9 (603.4–981.0)	9.0–100.5 (155.2–1732.8)	2.6 (44)	1.6–8.5 (27.6–146.6)	0.9–69.4 (15.5–1196.6)	<.001

Abbreviation: IMHA = immune-mediated hemolytic-anemia.

**Table 6:** Categorization of hyperbilirubinemia in dogs with and without neurologic signs.

Hyperbilirubinemia categories (mg/dL)	Number of dogs with neurologic signs (%)	Number of dogs without neurologic signs (%)	Odd ratios (95% CI)
**Mild (0.9–2.9)**	3/16 (19)	42/65 (65)	Baseline
**Moderate (2.9–5.8)**	5/16 (31)	15/65 (23)	4.7 (1.0–21.9)
**Severe (5.8–11.6)**	3/16 (19)	6/65 (9)	7.0 (1.1–43.0)
**Very severe (≥11.6)**	5/16 (31)	2/65 (3)	35.0 (4.7–262.6)

To convert bilirubin from mg/dL to μmol/L, multiply by 0.058.

**Figure 3:**
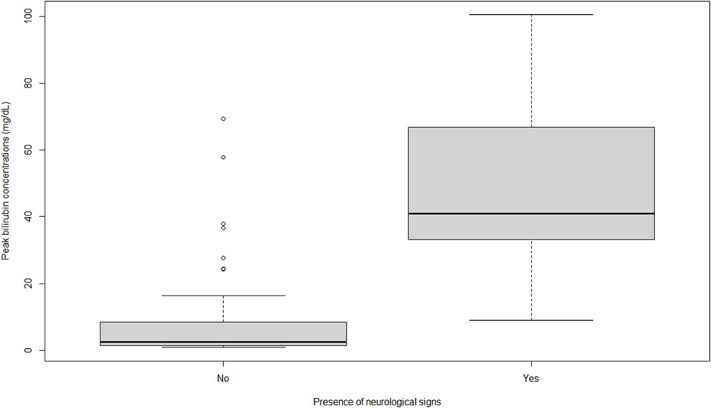
Boxplot showing the difference in peak bilirubin concentrations in 16 dogs with neurological signs and 65 dogs without neurological signs. Boxes represent the IQR from the 25th and 75th percentile. The horizontal bar in each box represents the median value. Outliers are represented by dots.

A strong association was found between peak bilirubin concentration and the presence of neurologic signs (*P* < .001; Table 5). The differences in peak bilirubin concentrations in dogs with and without neurologic signs are presented in Figure 3.

**Figure 4:**
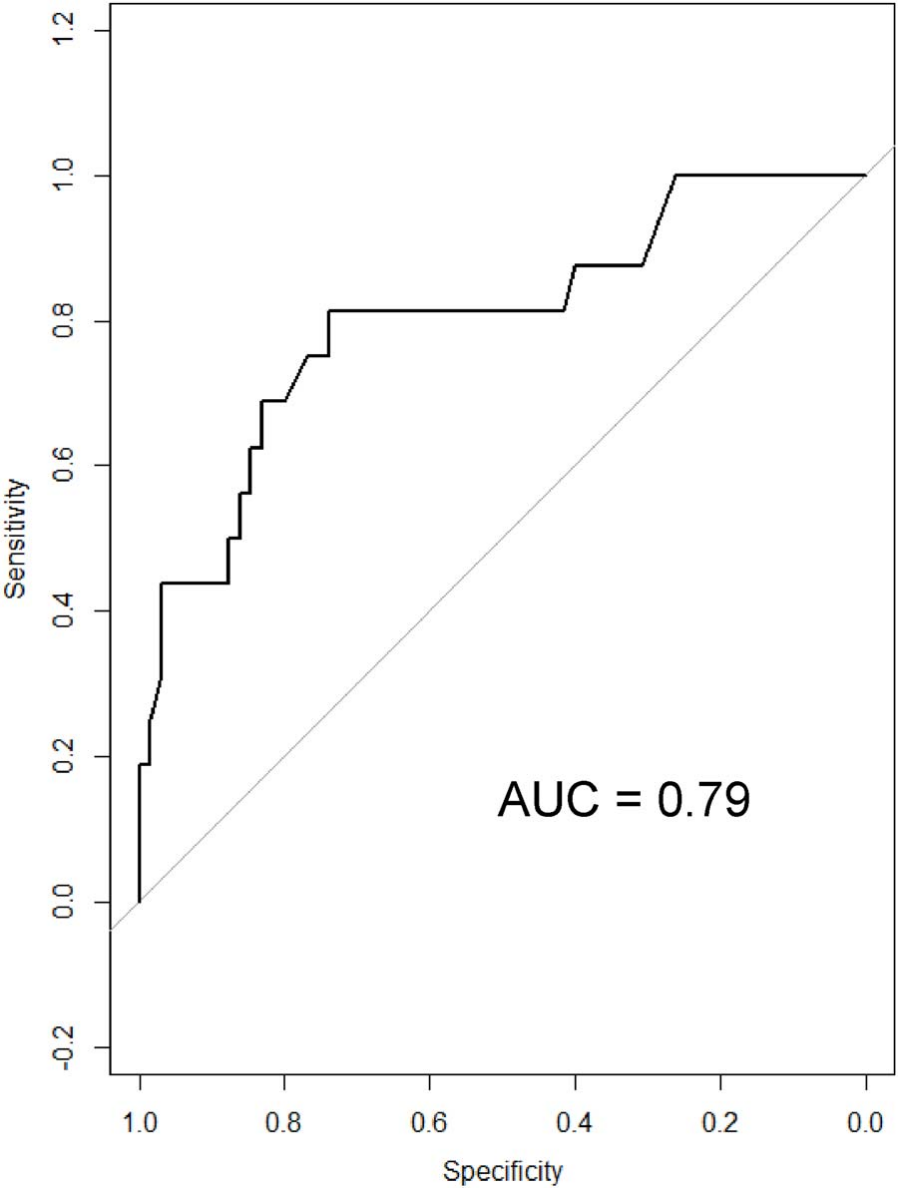
ROC curve discriminating between dogs with and without neurological signs in 81 IMHA dogs with hyperbilirubinemia (baseline bilirubin concentrations). Abbreviations: IMHA = immune-mediated hemolytic-anemia; ROC = receiver operating characteristic.

**Figure 5:**
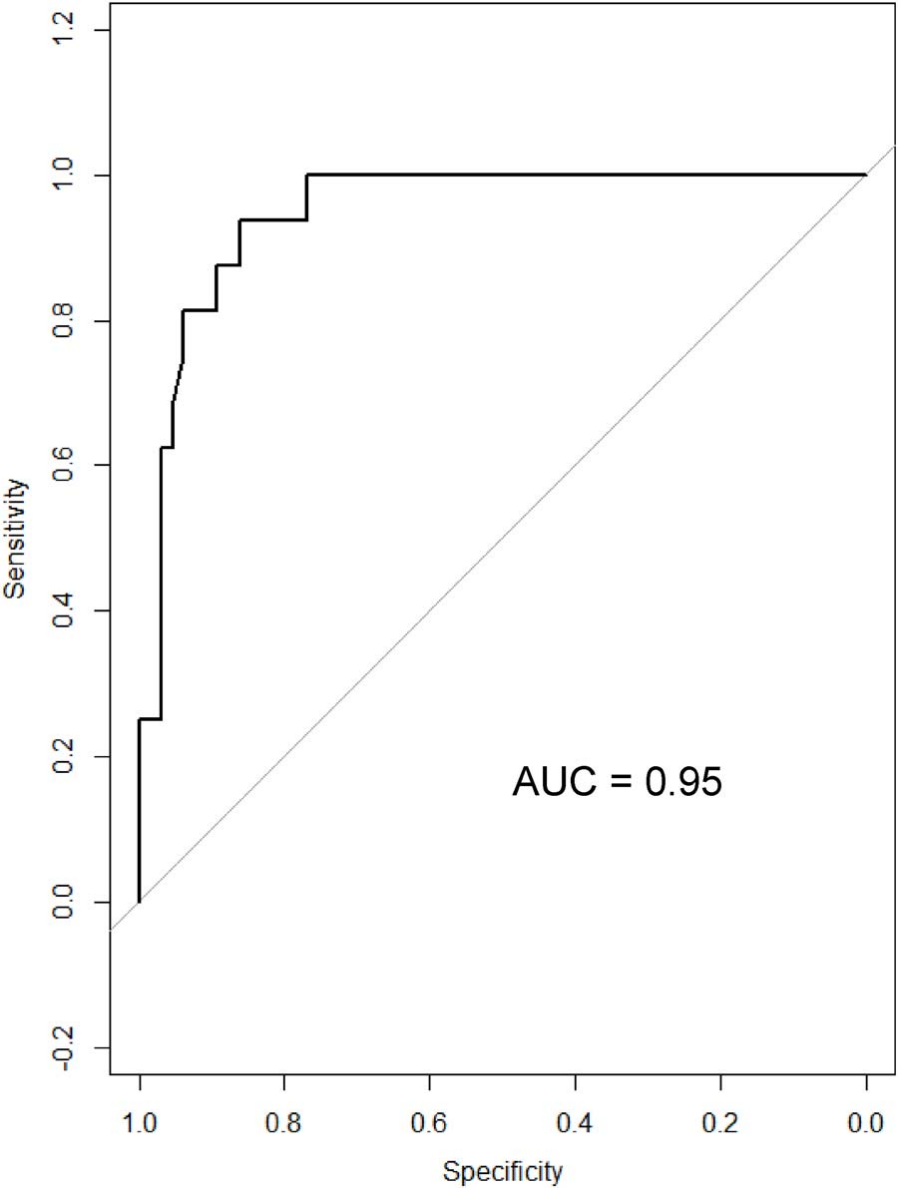
ROC curve discriminating between dogs with and without neurological signs in 81 IMHA dogs with hyperbilirubinemia (peak bilirubin concentrations). Abbreviations: IMHA = immune-mediated hemolytic-anemia; ROC = receiver operating characteristic.

The optimal bilirubin cut-off to discriminate between dogs with and without neurologic signs with sensitivity and specificity are reported in Table 7 and Figures 4 and 5. At an optimal baseline bilirubin cut-off of 3.3 mg/dL (≥57.5 μmol/L), a strong association was found between baseline bilirubin concentrations and the presence of neurologic signs (*P* < .001). The odds of developing neurologic signs were 12.2 (95% CI, 3.1-48.2) times more likely for dogs with baseline bilirubin concentrations ≥3.3 mg/dL (≥57.5 μmol/L) compared with dogs with baseline bilirubin concentrations <3.3 mg/dL (<57.5 μmol/L).

Similarly, at an optimal peak bilirubin cut-off of 13.9 mg/dL (239.5 μmol/L), a strong association was found between peak bilirubin concentrations and the presence of neurologic signs (*P* < .001). The odds of developing neurologic signs were 93.3 times (95% CI, 11-795.5) higher for dogs with peak bilirubin concentrations ≥13.9 mg/dL (≥239.5 μmol/L) compared with dogs with peak bilirubin concentrations < 13.9 mg/dL (<239.5 μmol/L).

The association between neurologic signs and other variables was assessed using univariable logistic regression modeling (Table 8). Dogs that took ≥2 days to reach peak bilirubin concentrations had higher odds of developing neurologic signs compared with dogs that took <2 days to reach peak bilirubin concentrations (*P* = .01). Conversely, dogs with higher BUN concentration at presentation were more likely to develop neurologic signs (*P* = .03). Dogs that were ≥7 years old had higher odds of developing neurologic signs (OR, 4.2; 95% CI, 1.1-16.1; *P* = .04).

Median bilirubin concentration at the time the neurologic signs developed was 39.2 mg/dL (675 μmol/L; IQR, 39.2–48.8; range, 9.0–99.5). Median bilirubin concentration when clinical improvement and resolution of neurologic signs were observed was 24.3 mg/dL (419.5 μmol/L; IQR, 12.0–37.6; range, 9.8–50.9) and 4.1 mg/dL (71 μmol/L; IQR, 3.4–14.1; range, 2.7–24.1), respectively. The median number of days from the onset of neurologic signs to clinical improvement was 1 day (IQR, 1–2; range, 1–3), with data available for only 5 animals. The remaining 11 dogs either died, were euthanized, or were referred to another facility. The median number of days until clinical resolution was 5.5 days (IQR, 3.5–7.8; range, 2–10), with data available for only 4 animals.

### Outcome

Fifty-eight (72%) dogs were discharged from the hospital, 18 were euthanized and 5 died. Reported reasons for euthanasia included financial concerns based on poor long-term prognosis (*n* = 7), respiratory distress (*n* = 5), central neurologic signs (*n* = 5), and sepsis and systemic inflammatory response syndrome (*n* = 1). Of the 5 dogs euthanized for respiratory distress, 3 had circulatory overload, and 2 had suspected pulmonary thromboembolism. Reported reasons for death included suspected pulmonary thromboembolism (*n* = 3) and acute cardiac arrest of unknown origin (*n* = 2).

Of the 16 dogs with neurologic signs, 5 survived (31%) and 11 were euthanized or died (69%). Reported reasons for euthanasia in this cohort included progressive neurologic signs (*n* = 5), financial concerns based on poor long-term prognosis (*n* = 4) and acute respiratory distress (*n* = 1). The cause for respiratory distress in 1 patient initially was suspected to be volume overload based on thoracic radiography findings, but pulmonary thromboembolism could not be excluded. The reason for death in 1 dog was acute cardiac arrest of unknown origin. In comparison, of the 65 dogs that did not develop neurologic signs, 53 survived (81%).

### Serial trends of hyperbilirubinemia

The median (IQR, range) bilirubin concentrations at baseline, peak, and decrease are reported in Table 9, along with the median number of days from baseline to peak, decrease, and normalization. Data for the initial decrease in bilirubin concentration were not available for 16 cases. Among these 16 cases, 2 dogs were discharged, 1 underwent tertiary referral and subsequently survived to discharge, and the remaining 13 died or were euthanized. Data for the normalization of the bilirubin concentration were available in 59 cases.

The median time from the first blood transfusion to peak serum bilirubin concentration was 1 day (IQR, 0–2; range, 2–8). A significant association was found between blood transfusion and peak bilirubin concentration (*P* < .001). The median peak bilirubin concentration was 1.4 mg/dL (24 μmol/L; IQR, 0.8–2.0; range, 0.9–36.9) for dogs that did not receive a blood transfusion and 5.6 mg/dL (96 μmol/L; IQR, 2.3–24.4; range, 4.1–100.5) for dogs that did receive blood transfusions. No association was found between the number of blood transfusions received and peak bilirubin concentration (*P* = .23).

## Discussion

Dogs with IMHA and increasing severity of hyperbilirubinemia had a higher likelihood of developing neurologic signs. In this cohort, the optimal baseline and peak bilirubin cut-off to identify dogs with neurologic signs were ≥3.3 mg/dL (≥57.5 μmol/L) and ≥13.9 mg/dL (≥239.5 μmol/L), respectively. Dogs with peak bilirubin concentrations ≥13.9 mg/dL (≥239.5 μmol/L) were 93 times more likely to develop neurologic signs, which suggests that these dogs may warrant more aggressive monitoring and treatment. Although clinicians should consider the potential development of neurologic signs when these bilirubin concentrations are exceeded, the cut-off values identified here are specific to our study cohort, and their generalizability to other populations of dogs with IMHA remains uncertain. Nevertheless, serial bilirubin measurements still might be clinically useful to guide appropriate treatment protocols and prognosticate outcomes for owners.

In humans, serum bilirubin concentration is a risk factor for developing ABE and secondary neurologic compromise.^20^ The etiology is multifactorial.^21^ In states of unconjugated hyperbilirubinemia, as is the case in IMHA, bilirubin crosses the blood–brain barrier and enters neuronal and glial membranes because of its lipid-soluble nature.^21^ In the brain, bilirubin has increased affinity for the globus pallidus, hippocampus, and subthalamic nucleus, where it is toxic to neurons.^21^ Although unconjugated bilirubin concentrations are therefore a predictive risk factor for the development of ABE, additional proposed mechanisms that contribute to its pathogenesis include excessive glutamatergic activity, disruption of the blood–brain barrier, decreased capacity for exportation of bilirubin, hypoalbuminemia, or a combination of these processes.^14^^,^^22^ None of the dogs in our study had hypoalbuminemia. Understanding the possible causes is critical for diagnosis and ensures appropriate treatment to minimize the likelihood of permanent neurologic dysfunction.

Immune-mediated hemolytic anemia is a complex, multisystemic disease with a wide range of pathophysiological processes that may independently or synergistically contribute to neurologic signs. Although dogs with increasingly severe hyperbilirubinemia had a higher likelihood of developing neurologic signs, we do not propose that bilirubin is the sole cause of neurologic signs in dogs with IMHA. The etiology of neurologic signs in dogs with IMHA is likely multifactorial, involving other contributing factors including systemic inflammation associated with a cytokine storm, hypercoagulability, the magnitude of anemia and resultant ischemia, the degree of hemolysis, and acid–base disturbances.^22^

In humans, the diagnosis of ABE is based on a combination of physical examination, clinicopathological, and neuroimaging findings. Key diagnostic criteria include an increased serum unconjugated bilirubin concentration (>19.8 mg/dL; >342 μmol/L),^20^ magnetic resonance imaging (MRI) abnormalities in the globus pallidus and subthalamic nucleus, and a high bilirubin-induced neurologic dysfunction (BIND) score.^21^ The BIND score is used to assess mental status, muscle tone, and crying patterns in newborns with hyperbilirubinemia. A higher score is associated with a worse prognosis.^21^

To date, no consensus exists regarding the diagnosis of ABE in dogs, and there is currently no reported use of a BIND score or analog in veterinary medicine. In our study, at an optimal peak bilirubin cut-off of ≥13.9 mg/dL (≥239.5 μmol/L) the sensitivity and specificity for identifying dogs with neurologic signs were 93.8% (95% CI, 71.7-98.9) and 86.2% (95% CI, 75.7-92.5), respectively. This cut-off is not specific in our cohort because other causes of neurologic dysfunction, such as intracranial vascular events or hypoxic brain injury, which are known to occur in dogs with IMHA, could not be excluded without advanced imaging or necropsy examination.^17^

**Table 7:** Optimal cut-off bilirubin concentrations to discriminate between dogs with and without neurologic signs with sensitivity and specificity.

Bilirubin	Optimal cut-off (mg/dL)	Sensitivity (95% CI)	Specificity (95% CI)	Power	Area under ROC (95% CI)	Odd ratio (95% CI)	*P*-value
**Baseline**	3.3	81.3 (57.0–93.4)	73.8 (62.0–83.0)	0.98	0.79 (0.67–0.93)	12.2 (3.1–48.2)	<.001
**Peak**	13.9	93.8 (71.7–98.9)	86.2 (75.7–92.5)	1.00	0.95 (0.90–0.99)	93.3 (11–795.5)	<.001
**Fold change**	2.0	93.8 (71.7–98.9)	73.8 (62–83)	1.00	0.85 (0.79–0.95)	42.4 (5.2–345.4)	<.001

To convert bilirubin from mg/dL to μmol/L, divide by 0.058.

One veterinary case report noted MRI findings consistent with those reported in the human medical literature,^14^ whereas another found no abnormalities despite substantial neurological compromise.^15^ In our study, no dogs with neurologic signs underwent advanced imaging. Instead, ABE was a leading differential diagnosis based on a substantially increased total bilirubin concentration, presence of neurologic signs, and the exclusion of other causes such as hepatic or renal insufficiency, or both. Advanced imaging should be considered in dogs with IMHA, severe hyperbilirubinemia, and neurologic signs to rule out ischemic or hemorrhagic stroke, intracranial tumors, or inflammatory encephalitis, which may present in a similar manner.

In our study, the highest recorded bilirubin concentration, likely representing the peak of the disease and the highest risk for the development of neurologic signs, occurred a median of 1 day after the first presentation. The time to initial decrease and normalization of the bilirubin concentration was much longer, at a median of 4 and 7 days, respectively. This observation suggests that IMHA is a rapidly progressive disease that can cause sustained hyperbilirubinemia, and a prolonged period of recovery should be anticipated.

**Table 8:** Univariable logistic regression of risk factors for neurologic signs in 81 IMHA dogs with hyperbilirubinemia**.**

Variable	Dogs with neurologic signs	Dogs with no neurologic signs	Odd ratios (95% CI)	*P*-value
**Categorical variables**	Number of dogs (%)	Number of dogs (%)		
**Baseline bilirubin (mg/dL)**	<3.3: 3 (6%) ≥3.3: 13 (43%)	<3.3: 48 (94%) ≥3.3: 17 (57%)	Baseline 12.2 (3.1–48.2)	<.001
**Peak bilirubin (mg/dL)**	<13.9: 1 (2%) ≥13.9: 15 (63%)	<13.9: 56 (98%) ≥13.9: 9 (38%)	Baseline 93.3 (11.0–795.5)	<.001
**Fold change (Peak/baseline bilirubin)**	<2: 1 (2%) ≥2: 15 (47%)	<2: 48 (98%) ≥2: 17 (53%)	Baseline 42.4 (5.2–345.4)	<.001
**Time to reach peak bilirubin (days)**	<2: 5 (10%) ≥2: 11 (37%)	<2: 46 (90%) ≥2: 19 (63%)	Baseline 5.3 (1.6–17.4)	.01
**Age (years)**	<7: 3 (9%) ≥7: 13 (28%)	<7: 32 (91%) ≥7: 33 (72%)	Baseline 4.2 (1.1–16.1)	.04
**Sex**	Female 8 (18%) Male 8 (22%)	Female 36 (82%) Male 29 (78%)	Baseline 1.2 (0.4–3.7)	.7
**Blood transfusion**	Yes 15 (21%) No 1 (10%)	Yes 56 (79%) No 9 (90%)	Baseline 2.4 (0.3–20.6)	.42
**Continuous variable**	Median (IQR, range)	Median (IQR, range)		
**Initial decrease in bilirubin (mg/dL)**	12.8 (11.0–33.6, 0.2–35.9)	1.0 (0.5–2.5, 0.0–55.2)	1.005 (1.001–1.008)	.02
**PCV at initial presentation (%)**	16.5 (14.8–21.2, 11–27)	18 (14.0–24.0, 6–33)	1.0 (0.9–1.1)	.62
**Creatinine (mg/dL)**	0.7 (0.5–1.0, 0.4–10.4)	0.7 (0.6–0.9, 0.01–1.6)	1.0 (0.99–1.03)	.4
**BUN (mg/dL)**	24.9 (20.0–39.8, 10.6–122.4)	19.6 (15.5–28.0, 8.4–79.8)	1.1 (1.0–1.2)	.03
**ALKP (U/L)**	168.0 (108.0–304.0, 8.0–689.0)	159.0 (110.0–20.07, 29.0–1828.0)	1.0 (0.998–1.003)	.7
**Neutrophils (X 10^9^/L)**	13.6 (9.3–19.6, 4.0–28.8)	15.5 (11.0–22.6, 1.5–145.0)	1.0 (0.9–1.0)	.27
**Monocytes (X 10^9^/L)**	1.4 (1.0–2.0, 0.3–4.8)	1.5 (1.0–2.5, 0.3–58.0)	0.9 (0.6–1.3)	.49

To convert bilirubin from mg/dL to μmol/L, divide by 0.058.

Abbreviations: BUN = blood urea nitrogen; IMHA = immune-mediated hemolytic-anemia; PCV = packed cell volume.

Our data identified that dogs that took longer to reach their peak bilirubin concentration were more likely to develop neurologic signs. This observation was unexpected, given that acute increases in bilirubin concentrations carry the highest risk for the development of ABE in humans.^20^ This finding suggests that kernicterus, or the chronic form of bilirubin encephalopathy, is a contributory factor in the development of neurologic signs in dogs and that sustained increases or prolonged exposure of the central nervous system to bilirubin may be more detrimental than previously thought. Alternatively, this finding could suggest that bilirubin encephalopathy was not the primary cause of neurologic signs in our cohort of dogs. Because of the retrospective nature of our study, each dog presented at different stages of the disease, potentially confounding this result.

Our data indicated a strong association between the administration of blood transfusions and peak bilirubin concentration, with a higher peak bilirubin concentration seen in dogs that received a blood transfusion. Cross-match incompatible blood transfusions may have contributed to these results, especially given that autoagglutination can interfere with commercial point-of-care cross-matching kits.^31^ In our study, all dogs were either cross-match compatible in the first instance or had cross-match compatibility testing within 72 hours of their first non-cross-matched transfusion.^29^ Although it is widely accepted that major crossmatching is not necessary for transfusion-naïve dogs, commercial crossmatching kits for dogs do not routinely evaluate minor compatibility, which could lead to undetected incompatibilities and an increased risk of hemolysis.^29^ Furthermore, the association between the administration of blood products and peak bilirubin concentration may reflect the severity of anemia and hemolysis, which prompted the transfusion. In our study, no association was found between the number of blood transfusions and peak bilirubin concentrations (*P* = .23).

Thirty-one percent (5/16) of dogs with neurologic signs survived to discharge. The outcomes associated with neurologic signs are likely multifactorial and difficult to evaluate retrospectively given that elective euthanasia is a confounding factor affecting mortality rate in our study. The overall survival rate was 72%, consistent with that previously reported.^2–7^

Creatinine, BUN, alkaline phosphatase (ALKP), as well as neutrophil and monocyte counts, were included in the statistical analysis because of their established association with the prognosis in dogs with IMHA.^28^ In our study, an increased BUN concentration was associated with a higher likelihood of developing neurologic signs, which, in turn, was associated with a worse prognosis. Increased BUN concentrations in IMHA have been postulated to occur secondary to pre-renal factors, hypoxia-induced renal injury, concurrent thromboembolic renal disease, or some combination of these.^32^ Although the retrospective nature of our study limits further exploration of the cause of increased BUN in these dogs with ABE, renal dysfunction leading to an accumulation of uremic compounds contributing to an encephalopathic state or a component of hypoxic brain injury are possible explanations. The higher likelihood of neurologic signs in dogs over the age of 7 years in our study was not unexpected, given the median age of presentation of IMHA in dogs is 6.5 years.^8^ Furthermore, increasing age has been associated with decreased blood–brain barrier function, potentially increasing the susceptibility of such dogs to neurologic dysfunction.^33^

**Table 9:** Serial trends of hyperbilirubinemia at baseline, peak, initial decrease, and normalization in 81 IMHA dogs.

		Bilirubin concentration mg/dL (μmol/L)	Number of days from baseline (days)
**Baseline**	Median	2.5 (43)	-
	IQR	1.4–4.8 (24.1–82.8)	-
	Range	0.9–37.3 (15.5–643.1)	-
**Peak**	Median	3.7 (64)	1
	IQR	1.8–24.4 (31.0–420.7)	0–2
	Range	0.9–100.5 (15.5–1732.8)	0–8
**Initial decrease^a^**	Median	1.1 (19)	4
	IQR	0.5–4.3 (8.6–74.1)	2–7
	Range	0–55.2 (0–951.7)	0–59
**Normalization^b^**	Median	-	7
	IQR	-	4–18
	Range	-	0–267

^a^Data for initial decrease in bilirubin concentration were not available for 16 dogs.

^b^Data for normalization (<0.9 mg/dL, <15 μmol/L) were available in 59 dogs.

Abbreviation: IMHA = immune-mediated hemolytic-anemia.

Of the 16 dogs with neurologic signs, only 1 underwent specific management for severe IMHA and suspected bilirubin encephalopathy. This patient underwent 3 rounds of TPE, experienced neurologic normalization, and survived to discharge. Therapeutic plasma exchange is an emerging extracorporeal treatment for immune-mediated disorders in veterinary medicine.^15^^,^^17^^,^^34^^,^^35^ Although immunosuppression remains the cornerstone of treatment for IMHA, TPE may be used to rapidly remove antibodies, immune complexes, and bilirubin until immunosuppression becomes effective.^15–17^^,^^35^ Although TPE has been used successfully in 2 dogs with IMHA and ABE,^15^^,^^16^ its benefit in comparison to medical management alone for IMHA without neurologic signs is unknown. Improved survival in dogs receiving at least 3 plasmapheresis treatments (92.9%) compared with those that received traditional medical management (61.8%) for IMHA has been reported^36^, whereas another study noted similar outcomes between dogs that received adjunctive TPE and those treated medically.^37^ The use of TPE is currently limited because of its high cost, limited availability, and the resynthesis of autoantibodies, often requiring multiple treatments.^35^ Future prospective studies should evaluate the efficacy of therapeutic interventions in the management of severe IMHA and hyperbilirubinemia in order to improve outcomes.

An important limitation of our study is that bilirubin encephalopathy cannot be confirmed based on clinical presentation alone, and no animals underwent advanced imaging of the brain or necropsy examinations to confirm the diagnosis. Neurologic examinations were not performed in all dogs, and furthermore, cases were not assessed by a board-certified neurologist. Currently, there is no consensus regarding neuroimaging findings in dogs with ABE, limiting our ability to confirm the diagnosis even if advanced imaging of the brain had been performed. Brain MRI was discussed with the owner in 2 of the 16 cases; but it was not performed because of concerns over the hemodynamic stability of the patient and high American Society of Anesthesiology grades. In clinical practice, advanced imaging is not always possible because of a combination of financial limitations, the acute and life-threatening nature of IMHA, and ethical concerns regarding the animal’s welfare. Animals with severe systemic diseases such as sepsis were excluded from our study, and we found no association between the presence of neurologic signs and PCV. Regardless, other causes of neurologic signs cannot be ruled out. Another limitation is the absence of diagnostic tests for vector-borne diseases. The dogs in our study were from southeastern Australia. As such, the prevalence of infectious diseases considered endemic in other geographic locations (eg, babesiosis) is low and explains the absence of testing for several vector-borne diseases in our study.^38^ The initial decrease of total bilirubin and time until normalization were not available for every case, and bilirubin concentrations were not measured daily or at standardized time points. It is therefore possible that the actual peak bilirubin was missed in some cases. The bilirubin severity grading system used in our study has not been previously validated or associated with patient centered outcomes in dogs, but its use has been reported in cats.^26^ Additionally, an interference of hemolysis on the bilirubin concentrations in those animals with intravascular hemolysis or ghost cells cannot be excluded despite the colorimetric diazo method being used. Adjustment for clustering by hospital was not performed because of the small number of hospitals and unbalanced case distribution. Repeated measures modeling of bilirubin concentrations was not conducted because of variable sampling intervals and incomplete serial data in our retrospective study. Because of the limited number of outcome events, only univariable analyses were performed.

Treatment protocols, including the type, dosage, and frequency of immunosuppressive treatment and thromboprophylaxis, were not standardized, nor were the hIVIG, TPE, or blood transfusion volumes. The timing of blood transfusions relative to the onset of neurologic signs varied in each dog, which limits the ability to determine an association between transfusions and neurologic signs and may introduce potential bias. Whether hIVIG and TPE improved outcomes in dogs is unknown, but both recently have been reported as successful interventions for IMHA and may have skewed the data.^16^^,^^39^ The dog that underwent tertiary referral for TPE only had bilirubin concentrations recorded before this intervention, making its impact on the results negligible. Because of the lack of long-term follow up in our study, there may be an under-estimation of the true outcome in these cases. Future studies should assess the long-term outcomes of dogs with IMHA presenting with hyperbilirubinemia and neurologic signs. Additionally, the number of transfusion reactions reported in our study may be partly explained by its retrospective design. Other potential causes of vomiting, diarrhea, or erythema, other than allergic transfusion reactions, could not be excluded. It also has been suggested that IMHA is associated with a higher rate of transfusion-related complications given the inherent pro-inflammatory state of the disease.^40^ Our rate of transfusion-associated reactions (24%) is similar to that previously reported.^40–42^ Additionally, a substantial proportion of dogs in our study received fresh or stored whole blood products (24%), despite pRBC being the preferred choice in euvolemic patients, which is often the case in IMHA.^11^ Although pRBC were utilized in 59% of reported transfusions, their use is often limited by accessibility.

### Conclusion

In our study population, hyperbilirubinemia was associated with the presence of neurologic signs, but bilirubin is unlikely to be the sole contributing factor. In our cohort, baseline and peak bilirubin cut-offs that discriminated between dogs with and without neurologic signs were identified, but these cut-offs should be interpreted cautiously because their applicability to other populations of dogs with IMHA has yet to be established. Our study further emphasizes that the presence of neurologic signs in dogs with IMHA is associated with higher rates of non-survival.

## Supplementary Material

aalaf034_Supplementary_table_S1

## Off-label antimicrobial declaration

The authors declare no off-label use of antimicrobials.

## Institutional animal care and use committee or other approval declaration

The authors declare no institutional animal care and use committee or other approval was required.

## Abbreviations

ABE: acute bilirubin encephalopathy
ACVIM: American College of Veterinary Internal Medicine
ALP: alkaline phosphatase
aPTT: activated partial thromboplastin time
BUN: blood urea nitrogen
CI: confidence interval
CT: computed tomography
DAT: direct antiglobulin test
IMHA: immune-mediated hemolytic anemia
pRBC: packed red blood cell
PCV: packed cell volume
PT: prothrombin time
RBC: red blood cell
SAT: saline autoagglutination test
TEG: thromboelastography
TPE: therapeutic plasma exchange
