# Supplementary material for: Hyperbilirubinemia and neurologic signs in dogs with non-associative immune-mediated hemolytic anemia: 81 cases (2015–2024)
Source: J Vet Intern Med. 2026 Jan 21;40(1):aalaf034. doi: 10.1093/jvimsj/aalaf034 (PMC12881973; doi:10.1093/jvimsj/aalaf034)
Supplement: aalaf034_Supplementary_table_S1 [file aalaf034_supplementary_table_s1.docx]

**Supplementary table S1.** Dog breeds

| Breed | Number of dogs |
| --- | --- |
| Mixed breed | 25 |
| Border Collie | 7 |
| Miniature Schnauzer | 6 |
| German Shepherd Dog | 4 |
| Maltese | 6 |
| Cocker Spaniel | 3 |
| Chihuahua | 3 |
| Dachshund | 2 |
| Irish Terrier | 2 |
| Lhasa Apso | 2 |
| Old English Sheepdog | 2 |
| Doberman | 2 |
| Australian Cattle dog | 2 |
| Airedale Terrier | 2 |
| Australian Kelpie | 1 |
| English Springer Spaniel | 1 |
| Finnish Lapphund | 1 |
| Fox Terrier | 1 |
| Italian Greyhound | 1 |
| Jack Russel Terrier | 1 |
| Japanese Spitz | 1 |
| Labrador Retriever | 1 |
| Pointer | 1 |
| Poodle (miniature) | 1 |
| Staffordshire Bull Terrier | 1 |
| Tibetan Terrier | 1 |
| Welsh Springer Spaniel | 1 |
